# Case Report: VV-ECMO rescue of severe pneumonia with polymicrobial mNGS detections and uncertain EBV significance in obesity

**DOI:** 10.3389/fmed.2026.1915648

**Published:** 2026-10-08

**Authors:** Fangrong Jiang, Ting Shen

**Affiliations:** Department of Critical Care Medicine, The Affiliated Yangming Hospital of Ningbo University, Yuyao, Zhejiang, China

**Keywords:** case report, Epstein–Barr virus, metagenomic next-generation sequencing, severe pneumonia, venovenous extracorporeal membrane oxygenation

## Abstract

Severe pneumonia with multiple microbial detections can make etiologic attribution and rescue decisions difficult. We report a 42-year-old man with obesity (body mass index, 36.3 kg/m^2^) who was admitted with rapidly progressive pneumonia, sepsis, acute hypoxemic respiratory failure, and multiorgan dysfunction [Sequential Organ Failure Assessment (SOFA) score, 9]. Despite invasive ventilation, one approximately 28-h prone-positioning session, and broad-spectrum antimicrobials, oxygenation deteriorated; immediately before extracorporeal support, PaO₂ was 52 mmHg on FiO₂ 1.0 (PaO₂/FiO₂, 52 mmHg). The course included blood-tinged secretions, suspected alveolar hemorrhage without a documented progressively bloody serial lavage pattern, and complete left lung atelectasis. Bronchoalveolar lavage fluid metagenomic next-generation sequencing (mNGS) detected Pseudomonas aeruginosa, Stenotrophomonas maltophilia, Legionella pneumophila, and a high Epstein–Barr virus (EBV) sequence burden. Admission blood cultures and conventional sputum culture showed no bacterial growth. On hospital day 3, venovenous extracorporeal membrane oxygenation (VV-ECMO) was initiated at 3,510 rpm, with blood flow 5.0 L/min and sweep gas flow 4.5 L/min. Adjunctive intravenous acyclovir (0.75 g every 8 h for 7 days) was administered because of the high EBV burden, although its clinical benefit in EBV pulmonary disease is unproven. VV-ECMO was discontinued on hospital day 18, extubation occurred on hospital day 24, and the patient subsequently returned to normal daily life. The clinical value of this case lies in management of a compound, potentially reversible phenotype rather than proof of any single microbial cause: severe pneumonia with polymicrobial mNGS detections, high EBV burden of uncertain significance, suspected hemorrhagic lung injury, unilateral atelectasis, and obesity-related respiratory mechanics successfully bridged with VV-ECMO.

## Introduction

Epstein–Barr virus (EBV), or human herpesvirus 4, is a ubiquitous herpesvirus that establishes lifelong latency and may reactivate during critical illness (1). Primary or organ-invasive EBV pneumonia is uncommon in adults; reported cases describe consolidation, nodules, ground-glass opacities, cavitation, and occasionally life-threatening respiratory failure (2–4). Because EBV DNA can be detected in respiratory specimens without proving tissue-invasive disease, respiratory EBV results require cautious interpretation alongside host immune status, conventional microbiology, other detected pathogens, and the overall clinical syndrome (16).

Diffuse alveolar hemorrhage (DAH) is a potentially fatal syndrome characterized by bleeding into the alveolar spaces, acute alveolar infiltrates, anemia or hemoptysis in some cases, and respiratory failure (5). Although immune-mediated vasculitis and connective tissue diseases are classic causes, infection-related alveolar hemorrhage has also been described in immunocompetent patients (6). In severe pneumonia, hemorrhagic alveolar flooding may rapidly worsen shunt physiology, impair secretion clearance, and limit lung recruitment.

Obesity further complicates acute respiratory failure because reduced functional residual capacity, impaired chest wall mechanics, and dependent atelectasis can amplify ventilation-perfusion mismatch in acute respiratory distress syndrome (ARDS) (7). When conventional measures such as lung-protective ventilation and prone positioning fail, venovenous extracorporeal membrane oxygenation (VV-ECMO) may provide a bridge for potentially reversible severe respiratory failure (8–11).

We describe an obese adult with severe pneumonia and polymicrobial mNGS detections, a high EBV burden in bronchoalveolar lavage fluid (BALF) of uncertain causal significance, suspected alveolar hemorrhage, complete left lung atelectasis, and refractory hypoxemia who was successfully rescued with VV-ECMO. The report focuses on transparent causal uncertainty and the practical role of extracorporeal support in a compound but potentially reversible hypoxemic phenotype.

## Case description

### Patient information and initial assessment

A 42-year-old man was admitted to the intensive care unit (ICU) on hospital day 1 because of dyspnea and somnolence for more than 1 day. His medical history comprised 4 years of treated hypertension; he had no known malignancy, organ transplantation, chronic infection, or exposure to corticosteroids, immunosuppressants, or chemotherapy. He was obese (height, 170 cm; weight, approximately 105 kg; body mass index, 36.3 kg/m^2^). He worked as a driver and had completed an approximately 14-h long-distance drive before symptom onset, but no relevant inhalational or other occupational exposure was identified. There was no family history of a similar illness or known inherited disorder and no relevant psychosocial history. Before admission, he self-treated fever with oral ibuprofen; no other treatment was received. Progressive dyspnea, somnolence, fatigue, occasional dry cough, blood-streaked sputum, and chills prompted presentation. Immediately before intubation, he was somnolent (Glasgow Coma Scale score, 14) and tachypneic at 40 breaths/min. His condition deteriorated in the emergency department, where endotracheal intubation and invasive mechanical ventilation were initiated before ICU transfer.

After intubation, the ICU admission temperature was 38.7 °C, heart rate 103 beats/min with a regular rhythm, respiratory rate 25 breaths/min, and blood pressure 128/62 mmHg; peripheral oxygen saturation was approximately 93% during mechanical ventilation. Examination showed widespread coarse moist rales bilaterally, no pathologic cardiac murmur or peripheral edema, warm skin without rash, petechiae, or ecchymoses, equal and reactive pupils, preserved brainstem reflexes, and no focal neurologic deficit. Laboratory testing showed leukocytosis (white blood cell count, 18.0 × 10⁹/L; neutrophils, 86.0%), lactate 4.3 mmol/L, procalcitonin 8.75 ng/mL, creatinine 137 µmol/L, alanine aminotransferase 54 U/L, aspartate aminotransferase 80 U/L, myoglobin 1,341.3 µg/L, and troponin 1.07 µg/L. An early post-intubation arterial blood gas obtained after emergency stabilization showed pH 7.36, PaCO₂ 42.8 mmHg, PaO₂ 106 mmHg, potassium 5.90 mmol/L, and sodium 128 mmol/L; the exact FiO₂ at that sampling was not reliably documented, so this PaO₂ value was not used to calculate the later nadir PaO₂/FiO₂ ratio. Chest computed tomography (CT) showed bilateral pneumonia with partial consolidation. Cranial CT showed no intracranial hemorrhage, acute or chronic infarction, or other structural lesion. Repeat chest CT during the initial assessment showed rapid progression, with extensive left lung consolidation and marked volume loss/atelectasis involving predominantly the left lower lobe and posterior upper lobe, together with mild right lower lung exudation.

The initial diagnoses were sepsis with vasopressor-dependent shock in the setting of presumed severe community-acquired pneumonia, acute hypoxemic respiratory failure, acute kidney injury, myocardial injury, liver dysfunction, and hyperlactatemia. The SOFA score was 9 on admission, 10 at VV-ECMO initiation, peaked at 12 during hospitalization, and was 6 at hospital discharge (Table 1). Only these verified aggregate scores were recoverable; component subscores were not imputed.

**Table 1:** Timeline of the main clinical events.

Relative time	Clinical event	Clinical significance
Hospital day 1	ICU admission with presumed severe pneumonia, sepsis with vasopressor-dependent shock, acute hypoxemic respiratory failure, and SOFA score 9	Substantial multiorgan dysfunction at presentation
Hospital day 1	Emergency-department intubation and invasive mechanical ventilation	Severe respiratory failure present before ICU transfer
Hospital days 1-2	Meropenem plus levofloxacin initiated for 14-day courses; organ support and one prone-positioning session lasting approximately 28 h	Optimized conventional rescue treatment; no further prone positioning after ECMO
Hospital day 3	Worst paired pre-ECMO gas: PaO₂ 52 mmHg on FiO₂ 1.0 (PaO₂/FiO₂, 52 mmHg); SOFA score 10	Refractory hypoxemia despite conventional treatment
Early course	Blood-tinged secretions predominantly from the left bronchial tree; progressive loss of left-lung ventilation	Suspected hemorrhagic lung injury and evolving complete left atelectasis
Hospital day 3	Bedside VV-ECMO via 17-Fr right internal jugular return and 21-Fr right femoral drainage cannulas	Extracorporeal gas-exchange support initiated
Hospital day 3	VV mode; 3,510 rpm; blood flow 5.0 L/min; sweep gas flow 4.5 L/min; circuit oxygen fraction 1.0	Enabled lung-protective ventilation and repeated airway clearance
During ECMO	Unfractionated heparin with APTT target 60-80 s	No major ECMO-related bleeding or thrombosis
Early hospitalization	Repeated bronchoscopy and BAL; admission blood cultures and conventional sputum culture showed no bacterial growth	Airway drainage and mNGS-based pathogen assessment; no culture isolate for susceptibility testing
Hospital day 5 onward	Enteral trimethoprim-sulfamethoxazole 1.92 g every 6 h initiated for 14 days; adjunctive IV acyclovir 0.75 g every 8 h was given for 7 days	Treatment broadened after mNGS while organism attribution, EBV causality, and antiviral benefit remained uncertain
Hospital days 8, 10, and 20	Serum EBV DNA declined from 6.123 × 10^3^ to 2.431 × 10^3^ and then <5.000 × 10^2^ copies/mL	Descriptive systemic trend during multimodal treatment; not directly comparable with BALF mNGS sequence reads
During hospitalization	SOFA score peaked at 12; only aggregate scores were recoverable	Component subscores were not imputed
Hospital day 18	VV-ECMO discontinued after 15 days	Successful decannulation without major bleeding or thrombosis
Hospital day 24	Extubation to high-flow nasal oxygen	Respiratory support de-escalated
Hospital day 25	Transition to conventional nasal-cannula oxygen; awake and tolerating oral intake	Marked clinical recovery
Hospital discharge/follow-up	SOFA score 6 at hospital discharge; subsequent complete functional recovery	Returned to normal daily life during follow-up

### Timeline

The principal diagnostic and therapeutic events are summarized in Table 1.

### Diagnostic assessment

Despite deep sedation, lung-protective mechanical ventilation, one prone-positioning session lasting approximately 28 h, airway clearance, bronchodilator therapy, initial fluid resuscitation, and vasopressor support, oxygenation deteriorated. No further prone positioning was performed after VV-ECMO initiation. Immediately before ECMO, the worst paired arterial blood gas showed PaO₂ 52 mmHg on FiO₂ 1.0, corresponding to a PaO₂/FiO₂ ratio of 52 mmHg; the earlier post-intubation PaO₂ of 106 mmHg was obtained at a separate time point. The maximum norepinephrine dose was approximately 0.26 µg/kg/min. Bedside echocardiography showed a left ventricular ejection fraction of approximately 65%; quantitative right ventricular function, estimated pulmonary artery pressure, and detailed valvular or pericardial measurements were not available in the retrieved report. Lung ultrasound showed residual A-line patterns adjacent to an irregular subpleural consolidation boundary (the shred sign) in the left lung, indicating heterogeneous preserved aeration next to consolidated, poorly aerated lung rather than a normal homogeneous A-line pattern.

During the early disease course, blood-tinged secretions increased and later became thin and watery. Bronchoscopy localized most secretions to the left bronchial tree and showed diffuse mucosal congestion without a discrete endobronchial lesion or active bleeding. In the context of repeated bronchoscopic assessment and airway lavage, the findings were considered compatible with suspected alveolar hemorrhage. However, progressively bloodier sequential BAL aliquots were not documented, and histopathology and hemosiderin-laden macrophage quantification were unavailable; therefore, definitive diffuse alveolar hemorrhage was not diagnosed. Ventilation of the left lung progressively decreased, culminating in complete left lung atelectasis and further oxygenation failure.

To identify pathogens and improve airway drainage, bronchoscopy with bronchoalveolar lavage was performed repeatedly. Blood mNGS detected Staphylococcus aureus, Legionella pneumophila, and Pneumocystis jirovecii. BALF mNGS detected Pseudomonas aeruginosa (3,080 sequence reads; relative abundance, 8.18%), Stenotrophomonas maltophilia (163 reads; 0.43%), Legionella pneumophila (91 reads; 0.24%), and human herpesvirus 4/EBV (13,742 reads; 36.48%). Admission blood cultures and conventional sputum culture showed no bacterial growth; consequently, no conventional isolate or antimicrobial susceptibility result was available. These organisms are therefore reported as mNGS detections rather than culture-confirmed causes. Serial serum EBV DNA values were 6.123 × 10^3^ copies/mL on hospital day 8, 2.431 × 10^3^ copies/mL on hospital day 10, and <5.000 × 10^2^ copies/mL on hospital day 20. These serum qPCR values describe a systemic trend but cannot be directly compared with BALF mNGS sequence reads because the specimen compartments and measurement methods differ.

Host immune evaluation did not identify chronic immunocompromise: HIV testing was negative; lymphocyte subsets, serum immunoglobulins, and complement levels were within reference ranges; and there was no history of steroid or other immunosuppressive therapy, cancer chemotherapy, or transplantation. Serum beta-D-glucan (G test) and galactomannan were negative. Antinuclear antibodies, antineutrophil cytoplasmic antibodies, anti-glomerular basement membrane IgG, anticardiolipin antibodies, IgG4-related testing, and PR3/MPO antibodies were negative. These findings reduce the likelihood of chronic immunodeficiency or immune-mediated hemorrhage but do not exclude transient sepsis-associated immune dysfunction.

The principal differential diagnoses were severe bacterial or atypical pneumonia with possible opportunistic infection; EBV pulmonary involvement versus bystander reactivation; immune-mediated or infection-associated alveolar hemorrhage; and cardiogenic pulmonary edema. The mNGS findings and parenchymal imaging supported infectious lung injury, the negative autoimmune evaluation argued against immune-mediated hemorrhage, and the preserved left ventricular ejection fraction argued against primary cardiogenic edema. Nevertheless, culture confirmation and the benchmark criteria for tissue-invasive EBV disease and diffuse alveolar hemorrhage were absent, so the final interpretation remained severe pneumonia with multiple mNGS detections, suspected hemorrhagic lung injury, and atelectasis rather than a single proven etiology.

### Therapeutic intervention

Empirical antimicrobial treatment was started immediately after ICU admission with meropenem 1.0 g intravenously every 8 h and levofloxacin 0.5 g intravenously once daily; both were continued for 14 days. On hospital day 5, after the mNGS results, enteral trimethoprim-sulfamethoxazole 1.92 g every 6 h was added for 14 days. Airway clearance and organ support continued. Because of the high BALF EBV burden and deterioration, intravenous acyclovir was used empirically at 0.75 g every 8 h for 7 days. The most recent serum creatinine measured before the first acyclovir dose was 109 µmol/L, and actual body weight was approximately 105 kg. In this obese patient, retrospective Cockcroft-Gault creatinine clearance was approximately 73 mL/min using ideal body weight (66 kg) and 90 mL/min using adjusted body weight (82 kg); actual weight was not used. Both estimates exceeded the product label's > 50 mL/min threshold, so renal function did not require extension of the 8-h dosing interval (18). The 0.75-g dose corresponded to 11.4 mg/kg by ideal body weight and 9.2 mg/kg by adjusted body weight. These estimates support the interval only, not the 0.75-g dose, antiviral efficacy, or a validated EBV regimen; the prospective dosing weight was not documented.

Because severe hypoxemia persisted despite optimized conventional management and the pulmonary injury was considered potentially reversible, bedside VV-ECMO was initiated on hospital day 3. A 17-Fr return cannula was placed in the right internal jugular vein and a 21-Fr drainage cannula in the right femoral vein. Initial circuit settings were VV mode, pump speed 3,510 rpm, blood flow 5.0 L/min, sweep gas flow 4.5 L/min, and circuit oxygen fraction 1.0. The drainage pressure and oxygenator model could not be recovered from the clinical record. Unfractionated heparin was used for systemic anticoagulation, targeting an activated partial thromboplastin time of 60-80 s, with serial monitoring of platelet count, hemoglobin, and bleeding or thrombotic complications. Lung-protective ventilation continued with tidal volume 4-6 mL/kg predicted body weight, plateau pressure <30 cmH₂O, and PEEP 6-8 cmH₂O. Daily lung ultrasound and chest radiography were used to monitor recruitment and resolution of pulmonary lesions.

Hemodynamic management included serial assessment of mean arterial pressure, central venous pressure, lactate, and central venous oxygen saturation, together with a restrictive fluid strategy after initial resuscitation. Nutritional support was estimated according to an ideal body weight of approximately 65 kg, with a target energy intake of 1,500-1,700 kcal/day and enteral nutrition plus protein supplementation.

### Follow-up and outcomes

After initiation of VV-ECMO and mNGS-informed antimicrobial treatment, oxygenation improved gradually, inflammatory markers declined, and norepinephrine was tapered and discontinued. White blood cell count, high-sensitivity C-reactive protein, and procalcitonin decreased over time, while PaO₂ and the PaO₂/FiO₂ ratio improved. During anticoagulation, activated partial thromboplastin time generally remained within or near the target range of 60–80 s., platelet count did not show progressive decline, and hemoglobin remained relatively stable without clinically significant ECMO-related bleeding.

As pulmonary infiltrates resolved and spontaneous respiratory capacity recovered, VV-ECMO was discontinued successfully on hospital day 18 after 15 days of support. Weaning was uneventful, without major bleeding or thrombosis. The patient remained on ventilatory support after decannulation and was extubated to high-flow nasal oxygen on hospital day 24, followed by conventional nasal-cannula oxygen on hospital day 25. He regained full consciousness, communicated normally, tolerated oral intake, and showed marked clinical recovery. During follow-up, he recovered completely and resumed normal daily life. Radiological evolution and dynamic clinical trends are shown in Figures 1, 2.

**Figure 1:**
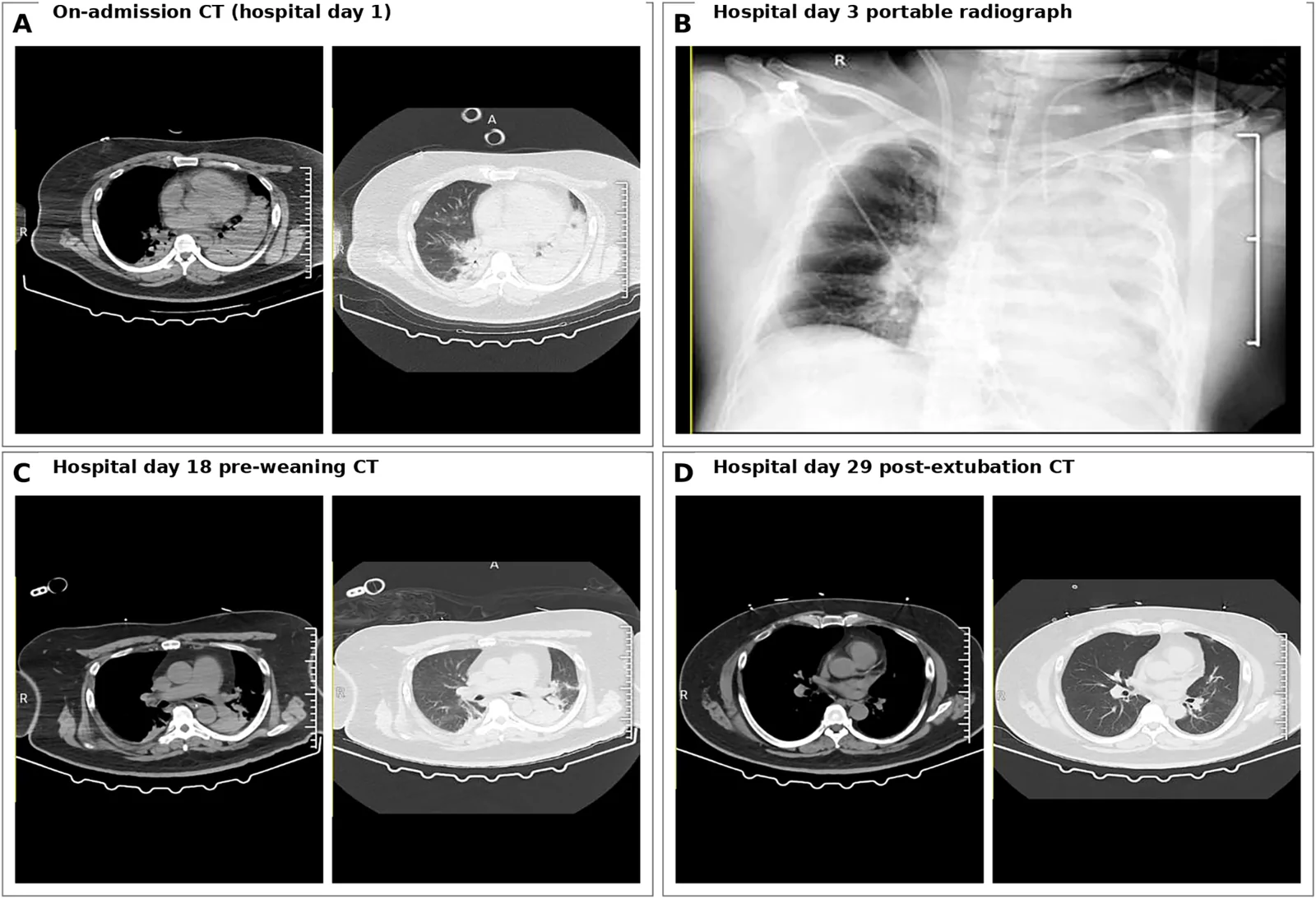
Radiological evolution during the episode of care. **(A)** On-admission CT (hospital day 1) shows extensive left-sided consolidation and volume loss. **(B)** Portable radiograph on hospital day 3, at VV-ECMO initiation, shows near-complete left hemithorax opacification. **(C)** Pre-weaning CT on hospital day 18 shows substantial re-expansion with residual dependent opacities. **(D)** Post-extubation CT on hospital day 29 shows near-complete radiological recovery.

**Figure 2:**
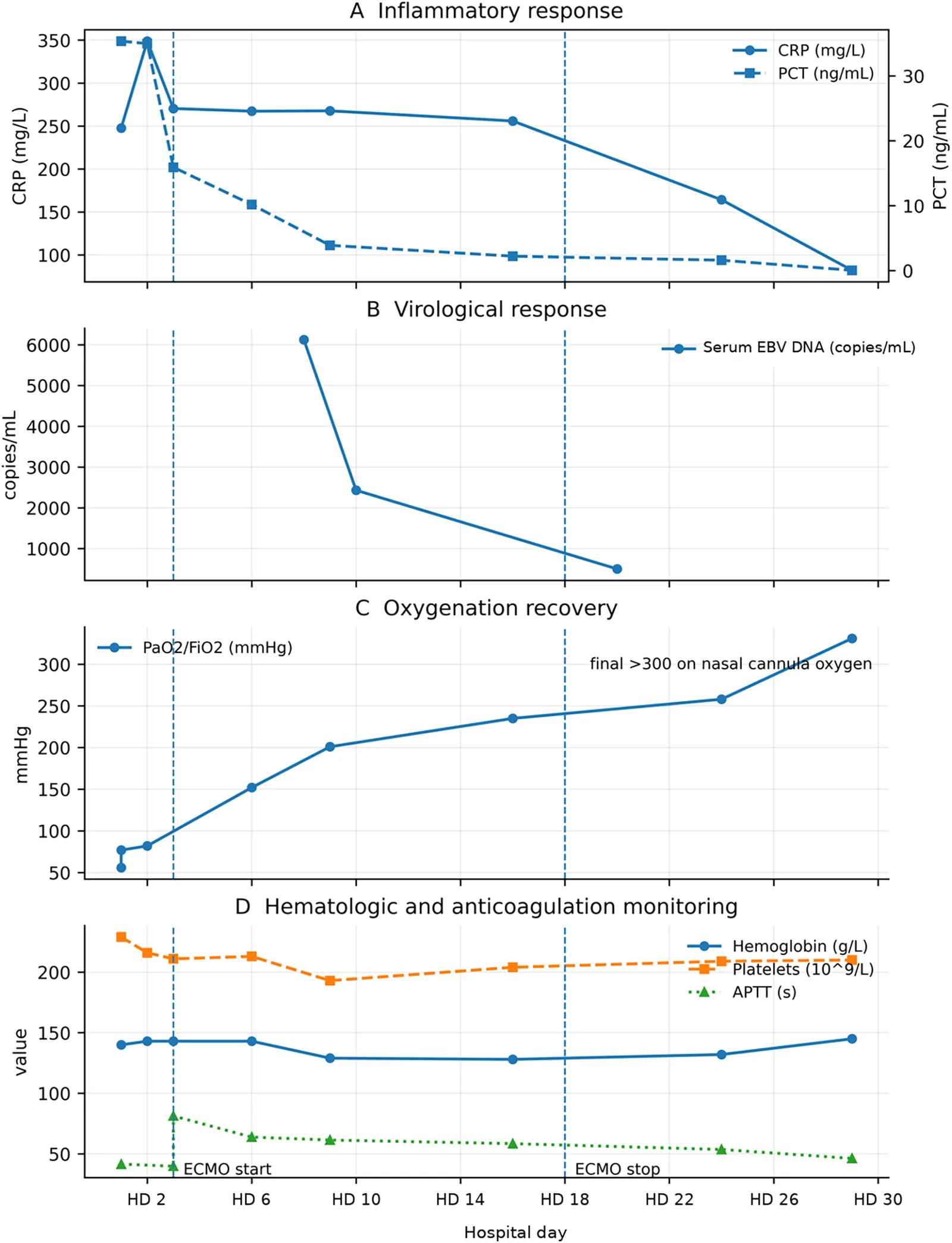
Dynamic clinical and laboratory trends by relative hospital day. (**A**) Serial C-reactive protein (CRP) and procalcitonin (PCT) values. (**B**) Serial serum quantitative Epstein–Barr virus (EBV) DNA values. (**C**) Serial PaO_2_/FiO_2_ ratios. (**D**) Hemoglobin, platelet count, and activated partial thromboplastin time (APTT) values during VV-ECMO support and recovery. Vertical dashed lines indicate VV-ECMO initiation on hospital day 3 and discontinuation on hospital day 18. Serum EBV DNA values are descriptive and are not directly comparable with BALF mNGS sequence reads because the specimen compartments and measurement methods differed. PaO_2_/FiO_2_ values during nasal-cannula support used an estimated FiO_2_ and are descriptive. The APTT target during unfractionated heparin anticoagulation was 60–80 s.

## Discussion

This case involved interacting contributors to severe respiratory failure: pneumonia and sepsis with polymicrobial mNGS detections, high respiratory EBV burden of uncertain causal significance, suspected hemorrhagic lung injury, unilateral atelectasis, and obesity-related respiratory mechanics. The report's novelty is not a claim that EBV or another detected organism caused fulminant pneumonia; it is the transparent interpretation and successful VV-ECMO rescue of a potentially reversible hypoxemic phenotype.

Adult EBV pneumonia has been reported in both immunocompromised and apparently immunocompetent patients, but EBV latency and reactivation make causal interpretation difficult (1–4). In a 2024 study of immunocompromised patients, EBV DNA was detected in more than one third of BAL samples submitted for qPCR, with limited direct impact on clinical diagnosis (16). Although this patient had no known chronic immunocompromising condition, HIV testing was negative, lymphocyte subsets and immunoglobulin levels were within reference ranges, and serum beta-D-glucan and galactomannan tests were negative, sepsis-associated transient immune dysfunction could still have enabled bystander EBV reactivation. The high BALF EBV sequence burden provides biological context but does not establish tissue-invasive EBV disease. The declining serum EBV DNA values are descriptive and cannot establish pulmonary causality or antiviral efficacy. Multiple bacterial, fungal, and viral mNGS detections, together with absent histopathologic confirmation, support the more defensible classification of severe pneumonia with polymicrobial mNGS detections and EBV as a possible co-factor or bystander rather than a proven cause.

Acyclovir inhibits lytic EBV DNA replication, but trials in infectious mononucleosis have not shown convincing, durable clinical benefit, and no controlled evidence establishes benefit in EBV pneumonia (17). Here, concurrent antimicrobial therapy, bronchoscopy, ventilation, and VV-ECMO preclude attributing recovery to acyclovir. The most recent serum creatinine before the first acyclovir dose was 109 µmol/L; retrospective Cockcroft-Gault estimates of 73 mL/min by ideal body weight and 90 mL/min by adjusted body weight indicated that renal function did not require extension of the 8-h interval (18). This supports the interval only, not the 0.75-g dose, antiviral efficacy, or a validated EBV regimen.

Suspected alveolar hemorrhage may have contributed to oxygenation deterioration. Diffuse alveolar hemorrhage can present with respiratory failure and diffuse infiltrates, and hemoptysis may be absent (5); infection-associated alveolar hemorrhage has also been described in immunocompetent hosts (6). Here, blood-streaked sputum, blood-tinged secretions predominantly from the left bronchial tree, diffuse mucosal congestion, negative autoimmune testing, and radiologic progression were compatible with hemorrhagic lung injury. Conversely, there was no active bronchoscopic bleeding, progressively bloodier sequential BAL pattern, histopathology, or hemosiderin-laden macrophage quantification. We therefore use the term suspected alveolar hemorrhage throughout and do not claim definitive diffuse alveolar hemorrhage.

Complete left lung atelectasis further worsened shunt physiology. In obesity, reduced functional residual capacity, increased chest wall load, and impaired respiratory mechanics predispose patients with severe pneumonia or ARDS to dependent collapse and recruitment failure (7). In this case, obesity likely lowered the threshold for atelectasis, while blood and inflammatory secretions may have promoted airway obstruction and poor ventilation of the left lung. This interaction explains why oxygenation remained critical despite high FiO₂ and prone positioning.

VV-ECMO was valuable as a bridge rather than disease-specific therapy. Prone positioning remains a key rescue strategy in severe ARDS (8), and ELSO guidance supports VV-ECMO for severe, potentially reversible respiratory failure refractory to optimized management (9). The EOLIA trial and subsequent meta-analysis support VV-ECMO in selected severe ARDS while emphasizing bleeding and other risks (10, 11). In this case, one approximately 28-h prone session did not prevent further deterioration. VV-ECMO at 3,510 rpm, blood flow 5.0 L/min, sweep gas flow 4.5 L/min, and circuit oxygen fraction 1.0 enabled less injurious ventilation, repeated bronchoscopy, and time for mNGS-informed antimicrobial treatment. Discontinuation after 15 days without major hemorrhagic or thrombotic complications supports carefully monitored ECMO use even when hemorrhagic lung injury is suspected.

Obesity should not be viewed as an absolute contraindication to VV-ECMO. Previous adult VV-ECMO studies and ELSO registry analyses indicate that increased BMI does not necessarily translate into higher mortality among patients receiving respiratory ECMO (14, 15). Nevertheless, obesity increases technical and physiological complexity, including cannulation difficulty, ventilation-perfusion mismatch, and recruitment challenges. In this patient, successful right internal jugular and right femoral cannulation, stable circuit flows, careful anticoagulation, and meticulous ventilatory and fluid management were probably essential to the favorable outcome.

This report has limitations. It is a single case and cannot establish causality between any mNGS detection and lung injury. Blood and conventional sputum cultures showed no bacterial growth, so no conventional isolate or antimicrobial susceptibility result was available; mNGS burden cannot distinguish infection from colonization, contamination, or reactivation without corroboration (12, 13). Definitive confirmation of EBV pneumonia or diffuse alveolar hemorrhage was unavailable. The follow-up EBV DNA measurements were obtained from serum by quantitative testing, whereas the initial respiratory EBV burden was measured as BALF mNGS sequence reads; these cross-compartment, cross-method values cannot be compared directly. Only verified aggregate SOFA scores were available at the reported time points; component subscores could not be reconstructed reliably and were not imputed. The exact FiO₂ for the early PaO₂ value of 106 mmHg, quantitative right ventricular and pulmonary pressure measurements, circuit drainage pressure, and oxygenator model were not recoverable retrospectively. The reported creatinine-clearance values are retrospective estimates rather than contemporaneously charted measurements, and the dosing weight selected prospectively was not recorded. Acyclovir was administered concurrently with broad antimicrobial and organ-supportive therapy, precluding attribution of clinical or virologic improvement to antiviral treatment. These limitations are central to the interpretation of the case.

## Patient perspective

During follow-up, the patient had recovered completely and returned to normal daily life. The patient and his family expressed appreciation that extracorporeal life support provided time for infection control and lung recovery.

## Conclusion

In this obese adult with severe pneumonia and polymicrobial mNGS detections, high BALF EBV burden of uncertain causal significance, suspected alveolar hemorrhage, complete left lung atelectasis, and refractory hypoxemia, VV-ECMO provided a successful bridge to mNGS-informed antimicrobial treatment and pulmonary recovery. EBV detection should not be equated with causal EBV pneumonia; it may instead represent a co-factor or bystander reactivation during sepsis. The take-away lesson is to combine careful microbiologic attribution, explicit diagnostic uncertainty, aggressive airway management, lung-protective ventilation, individualized anticoagulation, and timely VV-ECMO consideration for a reversible compound hypoxemic phenotype.

## Data Availability

The original contributions presented in the study are included in the article and its Supplementary Material. Further inquiries can be directed to the corresponding author.

## Supplementary material

SUPPLEMENTARY MATERIAL 1 Completed CARE Checklist for this case report.
