# Supplementary material for: Case Report: VV-ECMO rescue of severe pneumonia with polymicrobial mNGS detections and uncertain EBV significance in obesity
Source: Front Med (Lausanne). 2026 Oct 8;13:1915648. doi: 10.3389/fmed.2026.1915648 (PMC13687266; doi:10.3389/fmed.2026.1915648)
Supplement: Supplementary file 1 [file Supplementaryfile1.docx]

**COMPLETED CARE CHECKLIST**

**Manuscript ID: 1915648**

*Case Report: VV-ECMO rescue of severe pneumonia with polymicrobial mNGS detections and uncertain EBV significance in obesity*

**Reporting note.** Completed against the 2013 CARE Checklist. Section headings and figure/table labels identify where each item is addressed in the revised manuscript. Source: https://www.care-statement.org/checklist

| **Topic** | **Item** | **CARE checklist item** | **Reported in revised manuscript** |
| --- | --- | --- | --- |
| **Title** | **1** | The words ‘case report’ should appear in the title together with the area of focus. | Title |
| **Keywords** | **2** | Provide 2–5 keywords that identify the areas covered in the case report. | Keywords |
| **Abstract** | **3a** | Introduction—state what is unique about the case and what it adds to the scientific literature. | Abstract |
|  | **3b** | Describe the patient’s main symptoms and/or important clinical findings. | Abstract |
|  | **3c** | State the main diagnoses, therapeutic interventions, and outcomes. | Abstract |
|  | **3d** | Conclusion—state the main take-away lesson from the case. | Abstract |
| **Introduction** | **4** | Summarize why the case is unique, with reference to the relevant medical literature. | Introduction |
| **Patient information** | **5a** | Provide de-identified demographic and other relevant patient information. | Case description - Patient information and initial assessment |
|  | **5b** | Describe the patient’s main concerns and symptoms. | Case description - Patient information and initial assessment |
|  | **5c** | Describe medical, family, and psychosocial history, including relevant genetic information. | Case description - Patient information and initial assessment |
|  | **5d** | Describe relevant past interventions and their outcomes. | Case description - Patient information and initial assessment |
| **Clinical findings** | **6** | Describe the relevant physical examination and other significant clinical findings. | Case description - Patient information and initial assessment |
| **Timeline** | **7** | Organize important information from the episode of care as a timeline. | Table 1 - Timeline of the main clinical events |
| **Diagnostic assessment** | **8a** | Describe diagnostic methods, including physical examination, laboratory testing, and imaging. | Patient information and initial assessment; Diagnostic assessment |
|  | **8b** | Describe diagnostic challenges, including relevant limitations or barriers. | Diagnostic assessment; Discussion - limitations |
|  | **8c** | State the diagnosis, including other diagnoses considered. | Diagnostic assessment |
|  | **8d** | Report prognostic characteristics, such as staging, where applicable. | Diagnostic assessment |
| **Therapeutic intervention** | **9a** | Describe the types of intervention, such as pharmacologic, preventive, procedural, or supportive care. | Therapeutic intervention |
|  | **9b** | Describe administration of the intervention, including dose, strength, and duration where applicable. | Therapeutic intervention |
|  | **9c** | Describe changes in the intervention and provide the rationale for each change. | Therapeutic intervention; Table 1 - Timeline of the main clinical events |
| **Follow-up and outcomes** | **10a** | Report clinician-assessed and patient-assessed outcomes, when appropriate. | Follow-up and outcomes; Patient perspective |
|  | **10b** | Report important follow-up diagnostic and other test results. | Follow-up and outcomes; Table 1; Figure 2 |
|  | **10c** | Describe intervention adherence and tolerability, including how these were assessed. | Therapeutic intervention; Follow-up and outcomes |
|  | **10d** | Report adverse and unanticipated events. | Follow-up and outcomes; Discussion - limitations |
| **Discussion** | **11a** | Discuss the strengths and limitations of the approach to this case. | Discussion |
|  | **11b** | Discuss the relevant medical literature. | Discussion |
|  | **11c** | Provide the rationale for the conclusions, including an assessment of possible causes. | Discussion |
|  | **11d** | State the primary take-away lesson from the case report. | Conclusion |
| **Patient perspective** | **12** | Include the patient’s perspective or experience whenever possible. | Patient perspective |
| **Informed consent** | **13** | Confirm informed consent for publication and provide documentation if requested. | Ethics statement - written informed consent from the legal representative |

**Consent confirmation:** Written informed consent for publication was obtained from the patient's legal representative; see Ethics statement.
